# The impact of impending / onset of vision loss on depression, anxiety, and vision-related quality of life in Birdshot-Retinochoroiditis and Serpiginous Choroiditis

**DOI:** 10.1371/journal.pone.0239210

**Published:** 2020-10-05

**Authors:** Dominika Pohlmann, Anne Barth, Sergio Macedo, Uwe Pleyer, Sibylle Winterhalter, Özgür Albayrak

**Affiliations:** 1 Charité–Universitätsmedizin Berlin, Corporate Member of Freie Universität Berlin, Humboldt-Universität zu Berlin, Berlin Institute of Health, Berlin, Germany; 2 Department of Psychosomatic Medicine and Psychotherapy, Hannover Medical School, Hannover, Germany; 3 Department of Pediatric Cardiology and Intensive Care Medicine, Hannover Medical School, Hannover, Germany; Pusat Perubatan Universiti Kebangsaan Malaysia, MALAYSIA

## Abstract

To evaluate the impact of Birdshot-Retinochoroidopathy (BSRC) and Serpiginous Choroiditis (SC) on depression, anxiety, and vision-related quality of life. 72 individuals (BSRC: n = 28, SC: n = 8; healthy control group (HC): n = 36) completed the Patient Health Questionnaire-9 (PHQ-9), Generalized Anxiety Disorder-7 (GAD-7), and the Visual Function Questionnaire (VFQ-25). Multivariate linear regression models were used to analyze different subscales of the PHQ-9, the GAD-7 and the VFQ-25. The results showed that the mean of PHQ-9 was significantly higher while the mean of the VFQ-25 and its´ subscales were consistently lower in the disease group compared to HC. The mean of GAD-7 was not significantly lower in the disease group compared to HC. Stratification for different disease severity stages and duration of disease did not reveal any differences in sum scores of PHQ-9, GAD-7, and VFQ-25, whereas there were significant differences in some subscales of the VFQ-25. We conclude that BSRC and SC patients show higher levels of depression and a reduced visual quality of life due to imminent loss of vision. Because depression and quality of life are adversely affected by lack of social contacts and functioning, psychological treatment should enable patients to maintain their independence and ability to social interaction. Psychosomatic care should be taken in account for the treatment of BSRC and SC.

## Introduction

Birdshot-Retinochoroiditis (BSRC) and Serpiginous Choroiditis (SC) are rare forms of posterior uveitis (1–3%), which cause severe, chronic, progressive inflammation of both eyes and can lead to blurred vision and blindness [1, 2]. The prevalence of BSRC is higher in women, while men have a greater prevalence of SC [2]. Both diseases affect people at working age, causing significant economic, social, and psychological burden [3, 4].

Although BSRC and SC affect only the eyes, systemic immunosuppressive therapy (IMT) is required for these diseases, which can cause complications in other organ systems leading to a significant impact on quality of life [5]. Patients are exposed to blindness and adverse effects of IMT, leading to psychological and social problems. In order to assess the psychosocial situation of the patient, questionnaires have been established to examine potential problems. Assessment of health-related quality of life (HRQOL) is useful to examine physical and mental health aspects of an affected individual [6–8]. In brief, quality of life includes social functioning, mental health, and physical health and there are only few studies that assessed visual quality of life in ophthalmologic patients [9–11].

Moreover, the chronic and progressive nature of posterior uveitis with risk of blindness may place patients at greater risk for depression and anxiety. Only few studies have shown patients with various ocular diseases, which were screened for depression and anxiety by use of questionnaires [12–16]. Depression and anxiety have been determined as significant comorbidities among patients with severe ocular conditions such as glaucoma, age-related macular degeneration, and retinitis pigmentosa [14–16]. But there have been only limited reports on screening for psychological alterations in patients with uveitis [17–20].

We were particularly interested in BSRC and SC patients, who are at high risk of blindness during the course of their disease due to atrophy of the retina and macula. Therefore, we screened our patients with BSRC and SC for quality of life, depression, and anxiety and compared the results with an age and gender matched healthy control group. We were interested in: 1. the differences in the manifestation of symptoms of depression, anxiety, and of vision-related quality of life between BSRC, SC, and HC, 2. the expression of depression, anxiety, and vision-related quality of life depending on the duration and the severity of the disease, and 3. the correlation of vision-related quality of life with depression as well as with anxiety.

## Methods

### Patient recruitment

This cross-sectional study enrolled patients with BSRC and SC who were scheduled for an examination with multimodal imaging technique at Charité University of Medicine Berlin [21, 22]. Each patient was informed about the study and has given written consent. The patients were asked to complete the PHQ-9, GAD-7, and VFQ-25. Further we recruited a healthy control group from a general public matched for age and gender. This study was approved by the local ethics committee (EA4/055/16) of Charité Ethikkomission University of Medicine and follows the declaration of Helsinki.

### Questionnaires

We used the Patient Health Questionnaire (PHQ-9) to screen for symptoms of depression, the Generalized Anxiety Disorder 7 (GAD-7) to screen for symptoms of anxiety, and the National Eye Institute Visual Functioning Questionnaire 25 (VFQ-25) to examine vision-related quality of life.

#### PHQ-9

Symptoms of depression were assessed with the German version of the 9-item Patient Health Questionnaire-Depression Scale (PHQ-9) [23]. Each item is scored from 0 to 3, yielding a total score between 0 and 27. A total score ≥10 indicates the presence of a major depressive disorder (MDD). Cronbach’s α in the present study sample was 0.805.

#### GAD-7

Symptoms of anxiety were assessed with the German version of the 7-item Generalized Anxiety Scale (GAD-7) [24]. The items are also scored from 0 to 3, yielding a total score between 0 and 21. Cronbach’s α in the present study sample was 0.871. A cut-off of 8 implies the presence of generalized anxiety disorder, with a sensitivity of 92% and specificity of 76% for this diagnosis [25].

#### VFQ-25

The VFQ-25 examines vision-related quality of life caused by visual impairments or limitations. Emotional well-being and social functioning are captured by this questionnaire, as well as task-oriented areas, for example the ability to cope with every day-life situation. The questionnaire includes 25 items covering twelve subscales: “general vision”, “near and distance vision activities”, “ocular pain”, “vision-related social function”, “vision-related role function”, “vision-related mental health”, “vision-related dependency”, “driving difficulties”, “color vision”, and “peripheral vision” [26]. The VFQ-25 also contains a general health item. The reliability and validation of VFQ-25 has been proven to measure vision-targeted quality of life. The German translation of VFQ-25 has been validated in two studies [10, 27]. The self-administered form of the VFQ-25 was chosen, so that the patient would not feel pressured by the interviewer and would have sufficient time to complete the form. The evaluation of the VFQ-25 follows an algorithm calculation with scores ranging from 0 to 100. The higher the score, the better the functioning of vision and the higher quality of life. This study used the test manual methodology to generate one total score by averaging the twelve subscale values [26]. Cronbach´s α for the VFQ-25 total score was 0.958 and ranged from 0.679 and 0.927 across the twelve subscales.

### Statistical analyses

All statistical tests were performed with SPSS version 24. For the first aim (*differences in the manifestation of depression*, *anxiety*, *and vision-related quality of life between BSRC*, *SC*, *and HC*) the two-sample t-test was used. The analysis of variance (ANOVA) and/or Kruskal-Wallis-Test were used for the second aim (*expression of depression*, *anxiety*, *and vision-related quality of life depending on the duration and severity of disease*). For both tests a normal distribution of the population and variance homogeneity were prerequisite. The normal distribution was verified based on the Shapiro-Wilk test and variance homogeneity was verified using the Levene-test. For the third aim (*correlation of vision-related quality of life*, *depression and anxiety*) the Spearman- and Pearson-correlation was applied to correlate the subscales of the VFQ-25 and the total scores of the PHQ-9 and GAD-7. In addition, a multiple linear regression was utilized to analyze the impact of the different subscales of the VFQ-25 on depression and anxiety. The level of significance was set at α = p<0.05.

## Results

### Subjects

In total, 72 subjects (disease group: n = 36 (BSRC: n = 28, SC: n = 8); HC: n = 36) were examined and included for statistical analyses. HC was matched for age and gender (Table 1).

**Table 1:** Demographic characteristics.

n (%)	76 (100)
Healthy group (n, %)	36 (50)
Disease group (n, %)	36 (50)
BSRC (n, %)	28 (78)
SC (n, %)	8 (22)
Mean age in years (+/- SD, range)	62 (+/-10; 38–84)
Visual acuity **(**logMAR)	
BSRC (+/-SD, range)	0.4 (+/-0.6; 0–1.1)
SC (+/-SD, range)	1.1 (+/-0.8; 0–1)
BSRC	
Active	8 (29)
	12 (43)
Inactive, “burned-out”	8 (29)
SC	
Active	0 (0)
Inactive	8 (100)
Treatment of BSRC &SC (n, %)	
AD	8 (22)
AZA	1 (3)
CSA	7 (19)
MMF	12 (33)
MTX	2 (6)
IFN	2 (6)
Prednisolone <10mg	3 (8)

AD = Adalimumab; AZA = Azathioprine, BSRC = Birdshot Retinochoroiditis, CSA = Ciclosporine A; logMAR = Logarithm of Minimum Angle of Resolution; MMF = Mycophenolate mofetil; MTX = Methotrexate; IFN = Interferon; SC = Serpingiosa Choroiditis

### Differences in the manifestation of depression, anxiety, and vision-related quality of life between BSRC, SC, and HC

**Fig 1:**
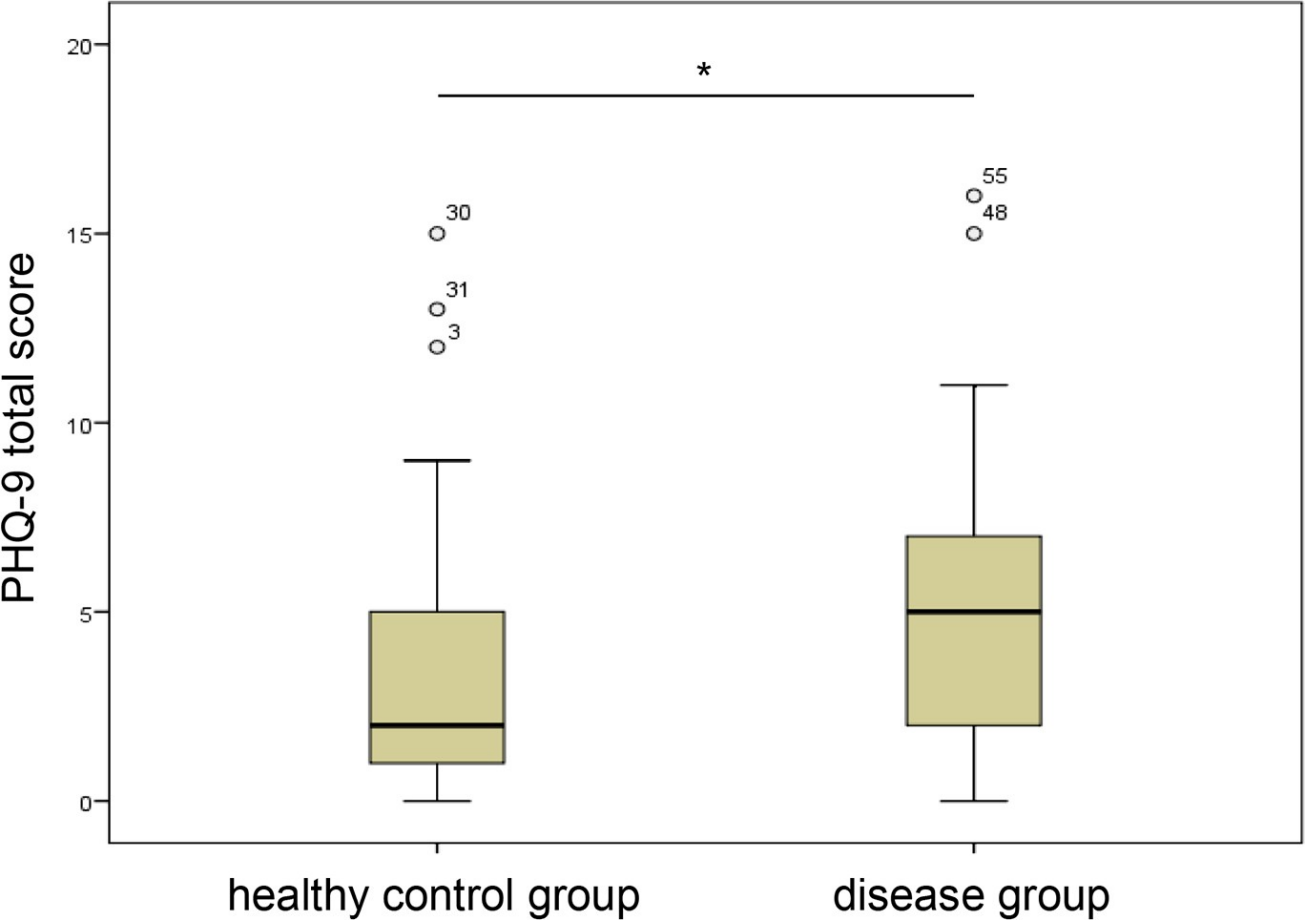
Differences in the manifestation of depression (PHQ-9 total score) in healthy control group and disease group. * p<0.05.

**Fig 2:**
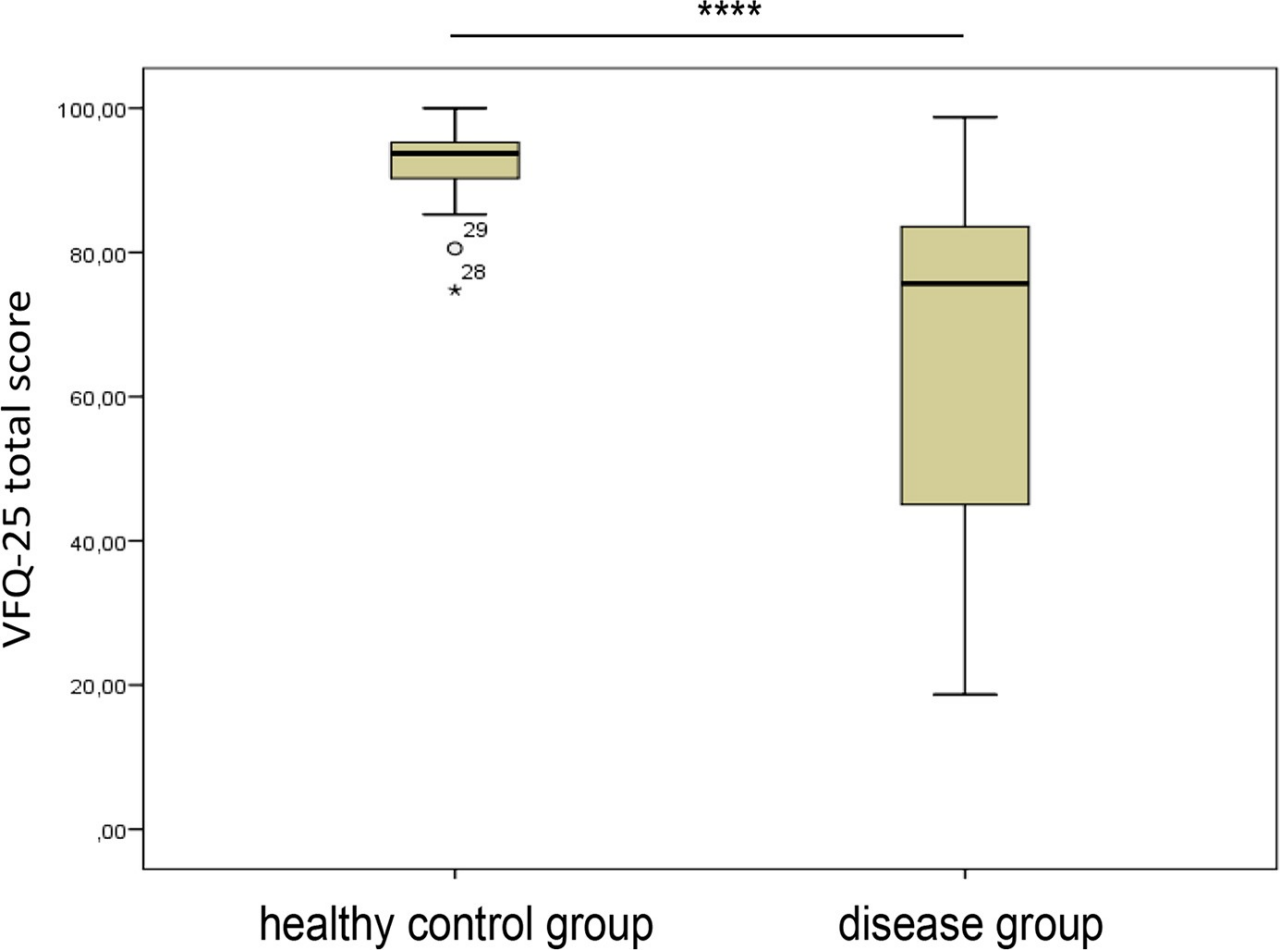
Differences in the manifestation of quality of life (VFQ-25 total score) in healthy control group and disease group. **** p<0.0001.

We hypothesized that differences can be found in the manifestation of depression, anxiety and vision-related quality of life between the disease group and HC. Our data showed differences in the manifestation of depression and vision-related quality of life between BSRC, SC and HC (Figs 1 and 2). There were significant differences for PHQ-9 total score and for VFQ-25 total score, as well as for all subscales of the VFQ-25 (Table 2), whereas no difference was found regarding GAD-7. The mean of PHQ-9 total score was higher while the mean of the VFQ-25 total score and its subscales were consistently lower in the disease group compared to HC. Thus, we confirmed our first hypothesis, showing higher levels of depression, but not anxiety, and lower levels for vision-related quality of life in the disease group compared to HC. There was no significance between the two disease groups.

**Table 2:** Comparison of PHQ-9 and VFQ-25 scores between disease group and healthy controls using the Mann-Whitney-U-test.

	Patients		Healthy controls		P value	Cohen d
	n	mean	n	mean		
PHQ-9 total score	36	42.08	36	30.92	**0.023**	0.554
GAD-7 total score	33	38.17	36	32.10	0.206	
VFQ-25 general health item	35	29.37	36	42.44	**0.003**	0.668
VFQ-25 general visual acuity	35	27.53	36	44.24	**<0.0001**	0.885
VFQ-25 ocular pain	36	31.64	36	41.36	**0.029**	0.478
VFQ-25 near vision	36	24.89	36	48.11	**<0.0001**	1.334
VFQ-25 distance vision	36	23.76	36	49.24	**<0.0001**	1.534
VFQ-25 vision-related social function	36	28.32	36	44.68	**<0.0001**	0.851
VFQ-25 vision-related mental health	36	22.81	36	50.19	**<0.0001**	1.731
VFQ-25 vision-related role function	36	21.89	36	51.11	**<0.0001**	1.950
VFQ-25 vision-related dependency	36	27.00	36	46.00	**<0.0001**	1.019
VFQ-25 driving difficulties	32	21.16	30	42.53	**<0.0001**	1.768
VFQ-25 color vision	36	29.00	36	44.00	**<0.0001**	0.768
VFQ-25 peripheral vision	36	26.15	36	46.85	**<0.0001**	1.138
VFQ-25 total score	36	22.47	36	50.53	**<0.0001**	1.806

### Expression of depression, anxiety, and vision-related quality of life depending on the duration and severity of disease

Secondly, we hypothesized that the expression of depression, anxiety and vision-related quality of life would deteriorate depending on the severity and disease duration in BSRC. The disease severity levels of BSRC were graded from “active” to “inactive”, and then to “inactive burned-out”. In the “active” group (n = 8), the mean age was 53.8 ± 10.5 years, and 62.5% were female. The “inactive” group (n = 12) consisted of 33.33% females with a mean age of 61.1 ± 8.6 years. The “inactive burned-out” group (n = 8) had 75% females and mean age was 68.5% ± 5.4 years. The SC group included only inactive disease, so that we focused on the BSRC group.

As for the severity of BSRC, no differences were found in the different subgroups of disease severity stage regarding PHQ-9 (p = 0.931), GAD-7 (p = 0.665), and VFQ-25 (p = 0.055) using Kruskal-Wallis-Test. To further specify this result, we considered the subscales of the VFQ-25 in our analysis. We could observe a decrease of the middle-ranking of the subscales of the VFQ-25 in patients with “inactive burned-out” disease stage. Small values of middle ranking indicate a low level of vision-related quality of life (Table 3).

**Table 3:** Results of the VFQ-25 subscales according to the Kruskal-Wallis-test, stratified by disease severity.

Scale	Chi-squared P value	Disease severity	Middle- ranking	Z value und P value (adjusted)	
General visual acuity	χ^2^ = 7.949 **P = 0.015**	Active	16.75	*Z* = 2.472 **P = 0.040**	
		Inactive	16.21		*Z* = 2.539 **P = 0.033**
		Inactive „burned-out“	7.07		
Distance vision	χ^2^ = 8.525 **P = 0.010**	Active	17.63	*Z* = 2.508 **P = 0.026**	
		Inactive	17.17		*Z* = 2.857 **P = 0.026**
		Inactive „burned-out“	7.38		
Social functioning	χ^2^ = 6.528 **P = 0.032**	Active	18.13	*Z* = 2.432 **P = 0.045**	
		Inactive „burned-out“	8.94		
Role difficulties	χ^2^ = 7.423 **P = 0.020**	Inactive	17.46	*Z* = 2.573 **P = 0.030**	
		Inactive „burned-out“	7.88		
Dependence on others	χ^2^ = 7.283 **P = 0.023**	Active	18.19	*Z* = 2.523 **P = 0.035**	
		Inactive „burned-out“	8.38		
Color vision	χ^2^ = 7.625 **P = 0.017**	Active	17,38	*Z* = 2.419 **P = 0.047**	
		Inactive	16.54		*Z* = 2.439 **P = 0.047**
		Inactive „burned-out“	8.56		
Peripheral vision	χ^2^ = 7.754 **P = 0.017**	Active	18.13	*Z* = 2.548 **P = 0.032**	
		Inactive „burned-out“	7.94		

Further, we analyzed PHQ-9, GAD-7 and VFQ-25 with respect to the disease duration. A disease duration of 0 to 2 years occurred in 4 patients, of which 50% were female with a mean age of 54.3 ± 14.3 years. Six patients had a disease duration of 3 to 4 years, of whom 50% were female and had a similar mean age of 55.8 ± 12.6 years. A disease duration of 5 to 10 years occurred in 14 patients, of whom 50% were female with a mean age of 62.5 ± 5.4 years. Four patients had a disease duration of 10 years or even more, of whom 75% were female with a mean age of 71.0 ± 5.5 years. Based on the total score no differences were found in the different disease groups according to the duration: PHQ-9 (p = 0.733), GAD-7 (p = 0.736), and VFQ-25 (p = 0.058) using Kruskal-Wallis-Test, unlike the subscales of the VFQ-25, where we calculated significant differences according to disease duration (Table 4).

**Table 4:** Results of the VFQ-25 subscales according to the Kruskal-Wallis-test, stratified by disease duration.

Scale	Chi-squared P value	Disease duration	Middle- ranking	Z value und P value (adjusted)
Near vision	χ^2^ = 8.816 **P = 0.021**	0–2 years	21.00	*Z* = 2.857 **P = 0.026**
		> 10 years	4.50	
Distance vision	χ^2^ = 8.816 **P = 0.021**	0–2 years	19.88	*Z* = 2.857 **P = 0.026**
		> 10 years	4.63	
Social functioning	χ^2^ = 8,816 **P = 0.021**	0–2 years	21.00	*Z* = 2.857 **P = 0.026**
		> 10 years	5.50	
Role difficulties	χ^2^ = 8.816 **P = 0.021**	3–4 years	19.33	*Z* = 2.857 **P = 0.026**
		> 10 years	3.88	
Dependence on others	χ^2^ = 8,816 **P = 0.021**	0–2 years	22.00	*Z* = 2.857 **P = 0.026**
		> 10 years	5.63	

In sum, we could not confirm our second hypothesis. However, as for disease duration, several aspects of vision-related quality of life seem to be impaired.

### Correlation of vision-related quality of life, depression, and anxiety

In our third hypothesis, we postulated that VFQ-25 would correlate significantly with the total scores of PHQ-9 and GAD-7. To verify this, we calculated the correlation coefficient according to Spearmen. Our data showed significant correlations between the subscales of VFQ-25 and PHQ-9 (Table 5), but not with GAD-7. To specify the relationship between vision-related quality of life and depression, we performed a multiple linear regression analysis including all subscales of VFQ-25. The model was significant (*F*(12,18) = 4.15, P = 0.003, R^2^ = 0.735, R^2^_corr_ = 0.558). This means that 55.8% of the variance of vision-related quality of life can be explained by depression. According to this result, subscales of the VFQ-25 can be used as a good predictor for the expression of depression [28]. In the second step of our linear regression model, the VFQ-25 subscale “mental health” was found to be significant (p = 0.001) (Table 6).

**Table 5:** Correlation coefficients, P values, and confidence measures for the relationship between the subscales scores of the VFQ-25 and the PHQ-9.

Scale	*r*_s_	*P*	*n*	*r*^2^
Distance vision	-0.357	0.032	36	0.127
Role difficulties	-0.345	0.039	36	0.119
Driving difficulties	-0.371	0.036	32	0.138
Mental health	-0.521	0.001	36	0.271
Dependence on others	-0.427	0.009	36	0.182
Peripheral vision	-0.461	0.005	36	0.213

**Table 6:** Multiple linear regression analysis for the prediction of VFQ-25 subscales for symptoms of depression.

VFQ-25 subscales	Regression coefficient β	T	P
Constant	15.163	4.368	<0.0001
General health	0.052	1.304	0.209
General visual acuity	0.026	0.543	0.594
Eye pain	-0.018	-0.548	0.591
Near vision	0.019	0.200	0.843
Distance vision	-0.012	-0.185	0.855
Social functioning	0.049	1.036	0.314
Mental health	-0.184	-3.772	**0.001**
Role difficulties	0.013	0.381	0.708
Dependence on others	-0.091	-1.645	0.117
Driving difficulties	-0.010	-0.424	0.677
Color vision	0.066	2.073	0.053
Peripheral vision	-0.034	-1.086	0.292

## Discussion

This study revealed the effects of impending or vision loss on depression, anxiety, and vision-related quality of life in vision threatening diseases. We found that BSRC and SC patients had higher levels of depression, and lower levels of vision-related quality of life, compared to HC. Duration of disease impaired several aspects of vision-related quality of life. The VFQ-25 subscale “mental health” positively predicted the occurrence of symptoms of depression in BSRC and SC patients compared to HC.

Patients with chronic somatic diseases have a high risk to display depression and anxiety [29]. In comparison to chronic somatic conditions, patients with reduced visual acuity even show higher levels of symptoms of depression and anxiety [30]. It is assumed that the deterioration of visual acuity, rather than the chronicity of the disease, leads to the manifestation of depression and anxiety in older people [30]. Since, in our study, the healthy control group was matched for age and gender, the impact of these parameters may be less likely to account for the differences in our study. In line with our findings, Augustin et al. did not find evidence for anxiety disorder in patients with age-related macular degeneration [31]. Moreover, Onal et al. found that 52.5% patients with uveitis screened for symptoms of anxiety were younger (Mean age: 31.5 vs. 40 years, p = 0.009) and had earlier onset of uveitis (Mean age: 26 vs. 35 years, p = 0.015) [17]. Since the mean age of the patients in this study was approximately 62 years, age might contribute to the missing significance of symptoms of anxiety in the disease group.

We found no significant differences in the expression of depression, anxiety, and vision-related quality of life in groups with various disease severity or disease duration, concordant to the work of Kuiper et al [32]. However, we found differences in the subscales of VFQ-25, that are relevant to everyday life. Both, BSRC and SC, are progressive conditions, with widely uncontrolled inflammation of the retina and the choroid, which leads to degeneration of photoreceptor layers and ultimately to blindness [21, 33]. While active inflammation with vasculitis and choroiditis usually occurs unnoticed, unless patients suffer from severe vitreous haze and macular edema, the “inactive burned-out” disease is more problematic due to retinal hypoperfusion and atrophy [21], which is reflected in our results. The “inactive burned-out” group showed decreased subscales of VFQ-25 in distance, peripheral, and color vision. In addition, the duration of inflammation is a risk factor for retinal and macular thinning with ellipsoid zone disruption [21], which is also reflected by our results. We observed significant differences of VFQ-25 subscales between a short disease duration (0–2 years) and a long disease duration (> 10 years). Comparable results were published, where patients experienced color blindness and limited peripheral vision at later stages of disease [34]. Both groups, respectively, showed differences in “role difficulties”, “social functioning”, and “dependence on others”. The vision-related quality of life in relation to these subscales was strongly related to the patient´s ability to cope with daily life. In line with this, Kempen et al. showed that impaired vision was associated with impairment in the ability to handle daily life activities [30]. Moreover, our results show that the progressive visual limitations do not limit activities of daily life at once, but rather were associated with a continuing decline. We conclude this from the fact that the middle-ranking of the subscales decrease continuously, rather than reaching a constant level after a short period of time, i.e. disease duration. Long-term studies would be needed, in order to verify our finding. Additionally, the subscale “dependence on others” should be considered in more detail in the future. Kempen et al. found that older persons (Mean: age: 77.4) with vision loss reported higher level of social support [30].

We demonstrated that subscales of the VFQ-25 predict the expression of symptoms of depression, especially the subscale “mental health”. This is somewhat trivial, since PHQ-9 records the expression of symptoms of depression, which in turn is a mental illness that negatively affects mental health. Therefore, it is self-explanatory that a total score that measures depression, as a mental limitation, correlates with the VFQ-25 subscale “mental health”. In addition, the VFQ-25 subscale “mental health” also captures the feeling of loss of control [35]. Lack of control over the feeling of worry is a sign of generalized anxiety disorder [36]. However, a significantly higher severity of generalized anxiety disorder in the disease group could not be observed. But we were able to show in our study that vision-related quality of life is related to “social functioning”, “role difficulties”, and “dependence on others”. It can be concluded that depression and quality of life are adversely affected by lack of social contacts and lack of social functioning. Thus, the focus of a treatment in BSRC and SC patients should be on maintaining the independence and ability for social interaction. Patients would benefit from psychological interventions that would support them to maintain their independence and help them to participate in public life and solve tasks of daily life, despite their visual impairment. Confidence in one´s own abilities should be maintained or restored.

The study has a few limitations. The sample size is small and makes it difficult to generalize the results. A multicenter study should be conducted to overcome the low incidence and prevalence of the diseases. Some of the items of the questionnaires were not completed, and we did not check for completeness.

In conclusion, our results demonstrated that BSRC and SC patients have a higher manifestation of depression and a reduced vision-related quality of life due to imminent loss of vision. No difference in the levels of depression, anxiety, and vision-related quality of life for the various disease severity and disease durations of BSRC could be recorded, but for some subscales of vision-related quality of life. “Mental health”, which is a predictor of depression should not be underestimated in the disease group. Therefore, a mental health worker specialized in psychosomatic care, should be involved. Early diagnosis of mental health disorders can improve the progression and prognosis of depression in BSRC and SC patients.

## Supporting information

S1 Data Anonymized data of the patients. (XLSX)
